# Spontaneous Unilateral Adrenal Hemorrhage in Pregnancy

**DOI:** 10.7759/cureus.977

**Published:** 2017-01-13

**Authors:** Salam Kadhem, Rawaa Ebrahem, Cyrus Munguti, Rami Mortada

**Affiliations:** 1 Internal Medicine, University of Kansas School of Medicine-Wichita; 2 Endocrinology, University of Kansas School of Medicine-Wichita

**Keywords:** spontaneous unilateral adrenal hemorrhage, pregnancy, spontaneous recovery

## Abstract

Spontaneous adrenal hemorrhage (SAH) is a serious medical condition associated with variable clinical presentation depending on the extent of the hemorrhage. Pregnancy-induced adrenal hemorrhage is poorly understood. A low cortisol level in the peripartum period with radiological findings is sufficient to establish the diagnosis. Prompt hormone replacement and supportive care to ensure good clinical outcomes is crucial. Due to the potentially life-threatening complications, physicians should have a high suspicion for adrenal hemorrhage when they evaluate patients with hypotension, fatigue, and abdominal pain during the peripartum period.

## Introduction

Abdominal pain is a common symptom in pregnancy and rarely is adrenal hemorrhage high on the list of differential diagnoses. Spontaneous adrenal hemorrhage (SAH) is an uncommon condition that if misdiagnosed can lead to adrenal crises [1]. SAH can occur in the absence of trauma or during the use of anticoagulants. The incidence of SAH ranges from 0.14% to 1.1%, and it usually involves the right gland [2]. The incidence during pregnancy is unknown [3]. While some patients with adrenal hemorrhage may be asymptomatic, the common presentation includes nonspecific pain located in the epigastrium, flank, upper or lower back, or pelvis in 65–85% of cases [2]. Symptoms of adrenal insufficiency, such as fatigue, weakness, dizziness, anorexia, nausea, vomiting, myalgia, and diarrhea, are present in approximately 50% of extensive, bilateral adrenal hemorrhage cases [1].

The most common causes of unilateral adrenal hemorrhage are blunt abdominal trauma, primary adrenal tumors, or metastatic tumors. Uncomplicated pregnancy infrequently has been associated with spontaneous unilateral adrenal hemorrhage [2]. We present a case of spontaneous unilateral adrenal hemorrhage occurring during pregnancy that was discovered after delivery and managed conservatively. Informed consent was obtained from the patient for this study.

## Case presentation

A 35-year-old female at 36 weeks of gestation presented with a one-week history of left flank pain, nausea, vomiting, and fatigue. She denied fever, urinary frequency, and urgency. The pain was sharp, constant, non-radiating, and gradually getting worse. She had been having this pain since the 24th week of gestation and had gone to the ER twice for the same reason. She had two previous uneventful pregnancies and no significant medical or surgical history. An examination revealed normal vital signs, a normal sized fundal height for gestation, and mild tenderness on the left renal angle.

**Figure 1:**
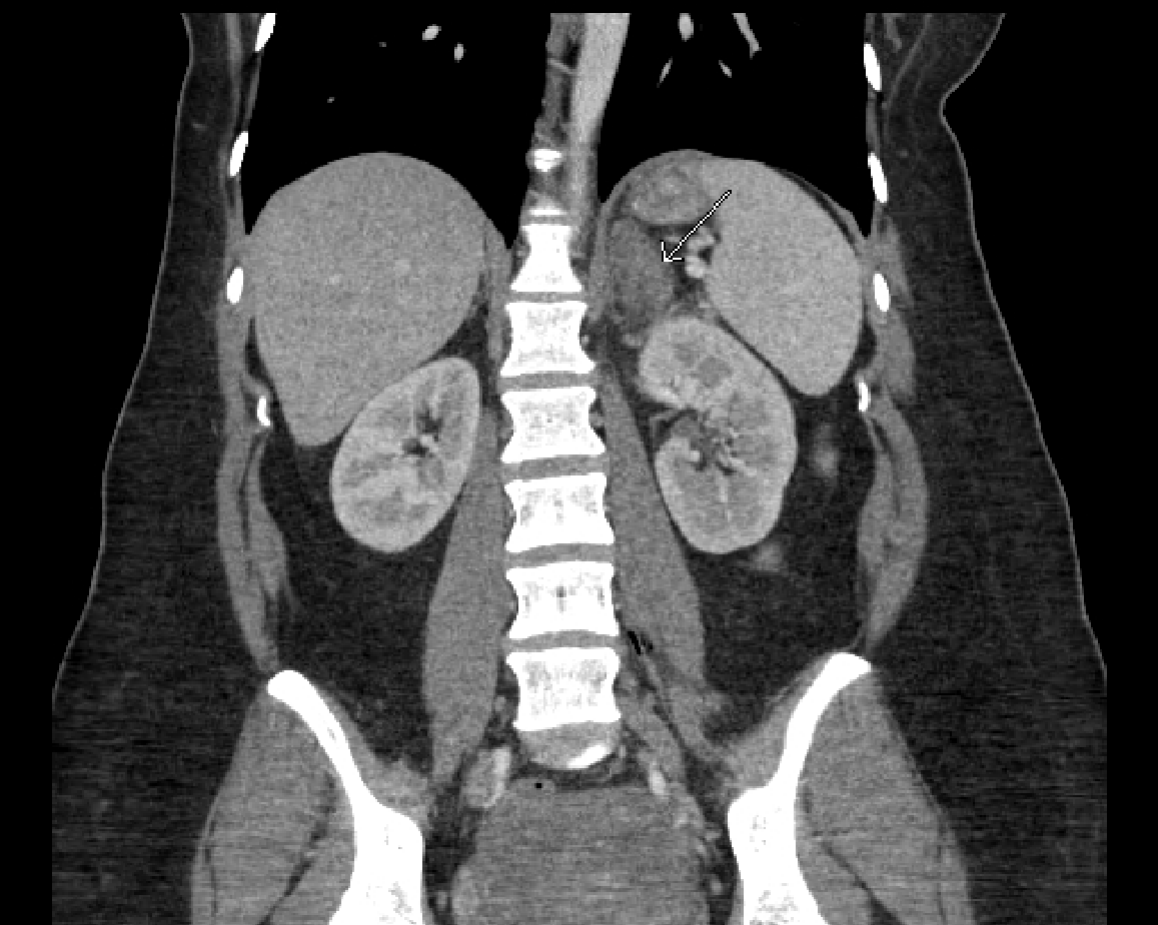
Sagittal CT scan image demonstrates left adrenal hyperattenuation (arrow) consistent with adrenal hemorrhage

**Figure 2:**
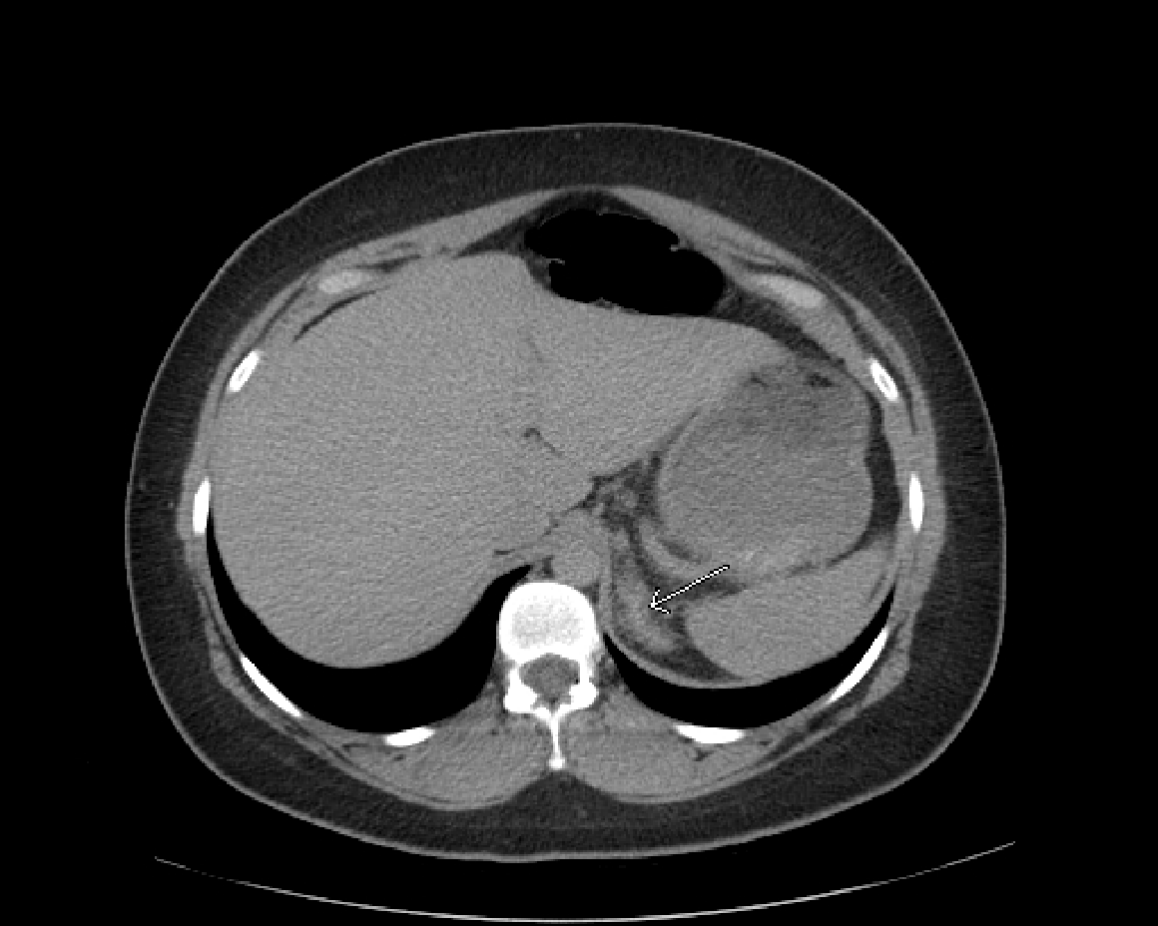
Cross-sectional abdominal CT scan with hyperattenuation of the left adrenal gland consistent with adrenal hemorrhage (arrow)

The urinalysis, complete blood count, and comprehensive metabolic panel were non-significant. An abdominal ultrasound was suspicious for adrenal bleed. She had a normal vaginal delivery on the second day of her hospitalization but continued to have left flank pain, as well as nausea, emotional lability, fatigue, and general malaise. A postpartum abdominal computed tomography (CT) scan revealed left adrenal hemorrhage (Figures 1-2).

Morning cortisol was low at 3.4 mcg/dl. She was started on oral hydrocortisone for spontaneous left adrenal hemorrhage, and her symptoms improved dramatically. She was discharged on oral steroids, and, during outpatient follow-up with endocrinology, she had no complaints, and her cortisol and adrenocorticotropic hormone (ACTH) levels were normal. A repeated CT scan of the abdomen showed resolving hematoma, and the steroid dose was tapered gradually and eventually stopped 15 months later.

## Discussion

Adrenal hemorrhage is an uncommon condition with a variable presentation that, if not recognized immediately and treated appropriately, may lead to acute adrenal crisis, shock, and death. The underlying mechanism of adrenal hemorrhage in non-traumatic cases is unclear; the available evidence has implicated ACTH, adrenal vein thrombosis, and spasm [4].

Adrenal hemorrhage can occur in pregnancy without apparent cause; however, adrenal hyperplasia during pregnancy increases arterial blood supply. Limited venous drainage might be a risk factor [5], and other differentials to be considered in pregnancy include pregnancy toxemia, twisted ovarian cyst, abortion, and postpartum hemorrhage.

Common presentations of adrenal hemorrhage include abdominal pain, tenderness, fever, hypotension, and shock, if not treated appropriately. Therefore, as previously mentioned, a high suspicion for adrenal hemorrhage with a low threshold for the diagnosis is crucial. Ultrasound is the first diagnostic choice during pregnancy, while MRI would be a highly sensitive as well as specific modality to rule out serious underlying causes like pheochromocytoma and adenoma [6].

Conservative management with fluid resuscitation, steroid therapy, and correction of underlying coagulopathies are necessary. Surgery is indicated for the patients who are not responding to conservative management and continue to deteriorate despite aggressive resuscitation. Adrenal insufficiency should be addressed to prevent circulatory collapse [7]. In severe hemorrhage, arterial embolization may be needed as a bridging for subsequent surgery [8]. Full recovery of adrenal function is expected following resorption of the hematoma, with discontinuation of steroid therapy [9].

## Conclusions

Non-traumatic adrenal hemorrhage is a rare but serious complication during pregnancy. Physicians should be aware of it and have a high suspicion and low threshold for this diagnosis due to the high mortality rate associated with improper treatment. Clinicians need to be aware of the variable presentations of SAH and prompt initiation of appropriate care. Steroid replacement is the key to a successful outcome.
